# The Arbuscular Mycorrhizal Symbiosis: Origin and Evolution of a Beneficial Plant Infection

**DOI:** 10.1371/journal.ppat.1002600

**Published:** 2012-04-19

**Authors:** Nicolas Corradi, Paola Bonfante

**Affiliations:** 1 Canadian Institute for Advanced Research, Department of Biology, University of Ottawa, Ottawa, Ontario, Canada; 2 Department of Life Science and Systems Biology, University of Torino, Torino, Piemonte, Italy; Duke University Medical Center, United States of America

## An Ancient and Ecologically Critical Fungal Lineage

**Figure 1:**
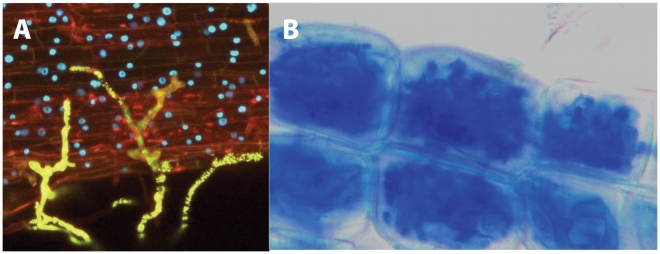
Establishment of the mycorrhizal symbiosis. An AMF contacts the surface of a legume root, by producing swollen structures called hyphopodia (in yellow) (A). Evident defence reactions are not detectable, and the epidermal cells appear alive, with the nuclei visible as blue spots. Once inside the root, the AMF colonizes the inner cortical cells, producing highly branched structures called arbuscules. Notwithstanding the massive colonization, the plant cells remain alive (B). Pictures kindly provided by Andrea Genre and Mara Novero, University of Torino.

Arbuscular mycorrhizal fungi (AMF) represent a monophyletic fungal lineage (*Glomeromycota*) that benefits terrestrial ecosystems worldwide by establishing an intimate association with the roots of most land plants: the mycorrhizal symbiosis. This relationship results in an improved acquisition of nutrients (e.g., phosphate and nitrates) from the soil by the plant partners and, in exchange, allows the AMF to obtain the photosynthetically fixed carbon sources (e.g., sugars) necessary for their survival and propagation [1], [2] (Figure 1). This fungal lineage is known to impact the function and biodiversity of entire ecosystems by producing extensive underground networks, composed of hyphae and spores, that interconnect a number of unrelated individual plant species [1], [2]. These networks also function as a significant sink for atmospheric carbon dioxide, and represent significant underground “nutrient highways” that benefit entire plant and microbial communities. Indeed, AMF spores and hyphae are also a valuable source of food for many soil microorganisms (i.e., bacteria, other fungi, and nematodes), and because of their many beneficial effects on terrestrial ecosystems, AMF are widely used in organic agriculture and plant nurseries to improve the growth of economically important species.

Besides their enormous benefits for terrestrial ecosystems around the globe, AMF are also known for their atypical evolutionary history and cellular features. For instance, it is currently thought that this intimate fungal–plant association has evolved over at least 500 million years—an extremely long-term co-evolutionary history, which has led many to suggest that AMF could have played a major role in the colonization of land by plants [3]. This hypothesis is also consistent with recent reports describing the capacity of some AMF species to infect the most ancient plant lineages (e.g., liverworts) and improve their overall fitness [4].

From a cellular point of view, AMF cells are at odds with those of many other eukaryotes, harbouring hundreds of haploid nuclei within one cytoplasm throughout their entire life cycle (i.e., septa are absent). The genetic structure of these co-existing nuclei has sparked a long-standing and intense scientific debate, and it is currently unclear whether such nuclei are genetically similar (e.g., homokaryons) [5] or divergent (e.g., heterokaryons) [6], [7]. Nevertheless, it is now generally accepted by the mycorrhizal research community that genes isolated from one spore are often characterized by an atypically high degree of intra-individual sequence polymorphism.

## The Elusive Genome of AMF Contains a Typical “Biotrophic” Gene Repertoire

Given the outstanding importance of AMF for the overall health and biodiversity of many ecosystems worldwide, one could easily assume that the genomes of many AMF would have been already sequenced and would be readily available in gene depositories for comparison and inspection. Unfortunately, however, this is far from being the case, and until earlier this year, sequence information on AMF consisted of only two published complete mitochondrial genomes and a handful of unrelated nuclear gene sequences. So, why is that? Obviously, there are many causes, the most notorious being difficulties in culturing these fungi under axenic conditions, the presence of a relatively elevated intra-individual sequence polymorphism hampering genome assemblies, and, finally, a nuclear genome size that could well be an order of magnitude larger than what was previously thought (e.g., most recent analyses suggest a genome size of around 150 megabases) [8], [9].

This past year, however, the field of AMF genome research experienced a major breakthrough with the publication of the transcriptome of the model AMF *Rhizophagus* (*Glomus*) *intraradices* [10]. The gene repertoire of this AMF was found to mirror that of other biotrophic fungi, with many genes involved in stealth host colonization and nutrient assimilation (e.g., metabolism of phosphate, nitrate, and lipids). Many AMF genes were also found to be shared exclusively with other, more diverged mycorrhizal fungi (e.g., *Tuber melanosporum*, an ascomycete, and *Laccaria bicolor*, a basidiomycete), providing long awaited insights into the origin and evolution of mycorrhiza-specific genes. The *R. intraradices* genome also encodes a large number of proteins that have not been reported in other genomes, suggesting that these have originated and been maintained to play an essential function exclusive to these ecologically relevant fungi [10].

## An Ancient Asexual Lineage with Many Genes Necessary for Sexual Reproduction

AMF have long been considered to represent an ancient asexual lineage, having evolved for over 500 million years in the absence of sexual reproduction. However, recent studies on the gene content of several species have revealed that these organisms harbour a battery of genes that generally function only during sexual processes [10], [11]. These include proteins that are known only through their involvement in the process of meiosis (e.g., *Spo11*, *Dmc1*, and *Rec8*) [12], as well as homologues of genes that compose the mating type locus of basal fungal lineages (e.g., *SexP* and *SexM* of Mucorales) [13]. The exact function of these gene sets is currently unknown, and, obviously, their identification is not conclusive evidence that AMF are indeed capable of undergoing some sort of cryptic sexuality. Nevertheless, the identification of sex-related genes in this supposedly ancient asexual fungal lineage opens up the exciting possibility that AMF may not represent one of the “highly exclusive” lineages commonly referred to as “ancient asexuals”, an artificial grouping that currently includes the AMF, the ostracod *Darwinula stevensoni*, and the bdelloïd rotifers [14].

## Mycorrhizal Colonization versus Pathogenic Infection: Similarities and Differences

As sessile organisms, plants have developed many strategies for interacting with microbes from different kingdoms, both beneficial and pathogenic, and a relevant goal in biology is to understand whether plant mutualists and pathogens share common molecular and cellular mechanisms for colonizing their hosts. Interestingly, a number of recent findings appear to support this possibility. A compelling example is represented by type III secretion systems, a molecular syringe that is used by both pathogenic and symbiotic bacteria to translocate effectors (i.e., secreted molecules that alter plant processes and facilitate colonization) into host cells [15]. The process for colonizing plant tissues reveals additional common aspects that are shared between fungal plant pathogens and symbionts. For instance, both rust fungi (pathogens) and AMF (symbionts) develop feeding structures surrounded by a membrane of host origin, and in both cases the physical separation of the fungus is complete but allows nutrient movements. In this particular example, sugars always flow from the plant towards the associated fungus, but only in the case of the AMF is the plant rewarded by a reverse flow of phosphate or nitrogen compounds [16].

The cellular and molecular mechanisms underlying the construction of these feeding structures also share many common aspects between fungal plant pathogens and symbionts. The biogenesis of the perihaustorial membrane typical of rusts requires complex polarized events of secretion [17] that mirror those found in the perifungal membrane biogenesis of AMF [18]. Accordingly, the transcriptomic profiles of haustorial and arbusculated cells show an impressive similarity, as their changes indicate an active metabolism in those cells directly involved in the response to the invading fungus, irrespective of its nutritional strategy. This has been shown using laser microdissection, which allows site-specific profiling specific to host processes following both types of interactions. For instance, in *Arabidopsis* infected by *Golovinomyces*, genes involved in photosynthesis, cold/dehydration responses, defence, auxin signalling, and cell cycle were detected [19], while similar analyses in arbusculated cells from legume plants revealed an activation of nutrient transporters, cell-wall-related genes, and transcription factors [18]. In both cases, the data pointed towards an enhanced plant metabolism imposed by both pathogenic and symbiotic fungi, and to an accommodation process related to their colonization events.

## Both Mycorrhizal and Pathogenic Fungi Have to Cope with the Plant Immune System

In order to deal with pathogens, plants have developed an innate immune system that triggers resistance mechanisms [20]. A dramatic increase in our current knowledge has originated from the characterization of both elicitors (or microbial-associated molecular patterns [MAMPs]) and effectors, the microbial molecules that initiate effector-triggered immunity [20]. Chitin is one of the best known fungal elicitors, and many chitin receptors have been identified as key regulators of plant responses [21]. In the case of AMF, it is quite interesting to observe that AMF release diffusible molecules that activate a range of responses in the host plant [22], and lipochitooligosaccharides have been recognized as one of these crucial elicitors [23]. The development of genomic sequencing projects for mycorrhizal fungi has opened unexpected possibilities: not only pathogenic fungi, but also mycorrhizal fungi produce effectors; as virulence factors they reach the host nucleus and activate different responses [24], [25]. These recent discoveries underpin how ecologically different organisms (e.g., pathogens versus symbionts) can use a very similar vocabulary during their dialogue with the host, suggesting that some of the determinants identified as modulators of host immunity are probably common to both types of associations [26].
